# Ferritin Heavy Subunit Silencing Blocks the Erythroid Commitment of K562 Cells via miR-150 up-Regulation and GATA-1 Repression

**DOI:** 10.3390/ijms18102167

**Published:** 2017-10-17

**Authors:** Fabiana Zolea, Anna Martina Battaglia, Emanuela Chiarella, Donatella Malanga, Carmela De Marco, Heather Mandy Bond, Giovanni Morrone, Francesco Costanzo, Flavia Biamonte

**Affiliations:** 1 Research Center of Advanced Biochemistry and Molecular Biology, Department of Experimental and Clinical Medicine, University Magna Græcia, 88100 Catanzaro, Italy; fabiana.zolea@alice.it (F.Z.); annamartinabattaglia@gmail.com (A.M.B.); fsc@unicz.it (F.C.); 2 Laboratory of Molecular Haematopoiesis and Stem Cell Biology, Department of Experimental and Clinical Medicine, University Magna Græcia, 88100 Catanzaro, Italy; emanuelachiarella@libero.it (E.C.); bond@unicz.it (H.M.B.); morrone@unicz.it (G.M.); 3 Department of Experimental and Clinical Medicine, University of Catanzaro “Magna Graecia”, 88100 Catanzaro, Italy; malanga@unicz.it (D.M.); cdemarco@unicz.it (C.D.M.)

**Keywords:** ferritin heavy subunit, differentiation, K562, miR-150, GATA-1

## Abstract

Erythroid differentiation is a complex and multistep process during which an adequate supply of iron for hemoglobinization is required. The role of ferritin heavy subunit, in this process, has been mainly attributed to its capacity to maintain iron in a non-toxic form. We propose a new role for ferritin heavy subunit (FHC) in controlling the erythroid commitment of K562 erythro-myeloid cells. FHC knockdown induces a change in the balance of GATA transcription factors and significantly reduces the expression of a repertoire of erythroid-specific genes, including α- and γ-globins, as well as CD71 and CD235a surface markers, in the absence of differentiation stimuli. These molecular changes are also reflected at the morphological level. Moreover, the ability of FHC-silenced K562 cells to respond to the erythroid-specific inducer hemin is almost completely abolished. Interestingly, we found that this new role for FHC is largely mediated via regulation of miR-150, one of the main microRNA implicated in the cell-fate choice of common erythroid/megakaryocytic progenitors. These findings shed further insight into the biological properties of FHCand delineate a role in erythroid differentiation where this protein does not act as a mere iron metabolism-related factor but also as a critical regulator of the expression of genes of central relevance for erythropoiesis.

## 1. Introduction

Megakaryocytic and erythroid progenitors that give rise to platelets and red blood cells, respectively, derive from a megakaryocyte-erythroid common precursor (MEP) [1]. The MEPs differentiation fate is dynamically determined by the coordination of different molecular events, including the progressive loss of differentiation potential, the expression of lineage-specific markers, and the acquisition of specialized morphological features and functionalities [1,2]. During erythroid maturation, MEPs undergo a progressive decrease in cell size, nuclear condensation, and activation of the transcription of globin genes with the consequent accumulation of hemoglobin [3]. Immunophenotypic studies also highlighted the acquisition and/or the increase in the expression of surface markers such as CD71 (Transferrin receptor protein 1, TfR1) and CD235a (Glycophorin A), now considered erythroid-specific hallmarks [4,5].

The MEPs fate is orchestrated by thecoordinated action of specific transcription factors. The dynamic exchange of GATA-1 with GATA-2, the so-called “GATA factor switch”, in which GATA-1 levels increase during the terminal erythroid maturation while GATA-2 is turned off, is one of the best-known examples [6,7].

This scenario is even more complex when taking into account the role played by microRNAs in the fine-tuning of haematopoiesis [8,9]. MicroRNAs are a class of small non-coding RNA whose activity as post-transcriptional inhibitor of gene expression belongs to the biological process, known as RNA interference [10]. Almost all of the stages of the haematopoietic lineage specification, from the maintenance of the haematopoietic stem cell pool to the generation of lineage-committed progenitors and of specific mature cells, are controlled by miRNAs [11]. Indeed, monocytopoiesis is regulated by miR-17/92 cluster while erythropoiesis is driven by miR-451, miR-16, and miR-144 and inhibited by miR-150, miR-155, miR-221, miR-222, and miR-223 [11]. MiR-125b supports myelopoiesis while B-cells maturation is promoted by the down-regulation of miR-34a [11]. Several reports indicate that miR-150 is involved in the control of multiple haematopoietic cell fates [12]. miR-150 is very highly expressed during advanced stages of both B and T cell maturation in bone marrow and thymus, respectively, and its premature expression leads to severe defects in B cell development through the down-regulation of target genes, such as *Myb* and *Foxp1* [13]. Within the myeloid lineage, a constant repression of miR-150 ensures the normal terminal erythroid development; on the contrary, its increased expression induces MEPs toward megakaryocytic maturation [14,15,16]. The role of miR-150 has been supported by several in vitro analyses: it has been shown that overexpression of miR-150 promotes the generation of colony-forming unit megakaryocyte (CFU-Mk), while its antagomiR-mediated suppression induces colony-forming unit erythrocyte (CFU-E) [17]; furthermore, forced expression of miR-150 significantly reduces hemin-dependent erythropoiesis, commitment to hemoglobinization and CD235a expression in the bipotent megakaryocyte/erythroid K562 human leukemia cells [18]. K562 cells can be terminally differentiated in vitro toward the erythroid and megakaryocytic lineages; thus, they are considered as a useful in vitro model for studying MEP commitment [1,2]. The molecular mechanisms underlying the effects of miR-150 on MEPs fate-decision are not fully elucidated. Different models have been proposed either associated with differentiation-related or proliferation-related pathways [15]. Moreover, gene expression profiling suggests that forced miR-150 expression in hemin-induced K562 cells suppress the activation of ErbB-MAPK-p38 and ErbB-PI3K-AKT pathways [18]. However, the upstream regulators of miR-150 have not yet been determined.

The MEPs function and fate are also affected by metabolic perturbations [19,20,21]. In particular, iron metabolism and erythropoiesis are intimately linked. An adequate supply of iron is indeed necessary to ensure sufficient hemoglobin synthesis and thus for the correct maturation of red blood cells [20,21]. However, an excessive amount of intracellular free iron may be harmful to the cells since it can trigger the generation of reactive oxygen species (ROS) through the Fenton reaction [22]. Ferritin, the main intracellular iron storage protein, tightly regulates iron levels by storing it in a nontoxic and bioavailable form for supply upon metabolic requirement of hemoglobinization [23]. Ferritin is a multimeric protein composed of a total of twenty-four subunits of two types, the ferritin heavy subunit(FHC, FTH) and the ferritin light subunit (FLC, FTL), assembled to form a shell that is able to sequester up to 4500 iron atoms [19,20]. FHC has a ferroxidase activity through which it converts Fe(II) to Fe(III) and protects cells against oxidative stress [24,25]. Indeed, we have recently demonstrated that FHC-silencing results in a significant increase in intracellular ROS in erythroleukemia K562 cells [25] as well as in other cell types [26]. At the same time, a growing body of experimental evidence has shed light on new and intriguing roles for FHC in the control of proliferation and migration of several cancer cell lines as well as in the regulation of many oncogenes and oncomiRNAs [24,25,26,27].

The role of FHC in the haematopoietic differentiation has been so far mainly explored in relation to its function in the iron intracellular metabolism. To date, the gene expression profiling after the hemin-mediated erythroid differentiation of K562 cells highlighted the occurrence of both transcriptional and translational up-regulation of the ferritin gene [23,28]. This results in an increase in ferritin synthesis that ultimately enhances the cellular capacity of iron storage for hemoglobin synthesis [23].

In this study, we investigated the role of FHC in K562 cells erythroid differentiation by exploring the effects of the perturbation of its intracellular amount on cell morphology, expression of representative genes and lineage-specific markers. Our results revealed that FHC knock-down induced a significant arrest in the erythroid commitment of K562 cells that was mostly mediated by the up-regulation of miR-150 and the parallel repression of GATA-1, and uncovers a new role of FHC in the lineage choice of the erythro-megakaryocytic K562 cells through the fine tuning of key regulatory molecules.

## 2. Results and Discussion

The K562 leukemia-derived cell line represents a useful in vitro model of MEP since they are situated at the common progenitor stage of erythroid and megakaryocytic lineages differentiation [1,2], and can be induced toward either of the above cell fates by a number of chemical agents, such as hemin and phorbol 12-myristate 13-acetate (PMA), respectively [29].

Ferritin is the main iron storage protein within the cell and is localized in cytoplasm, nucleus, and mitochondria [27]. The erythroid differentiation is accompanied by an enhanced expression of its heavy subunit (FHC), which has a ferroxidase activity, and this induction has been mainly attributed to the necessity for the cell to store in a non-toxic form the high amounts of iron required for optimal hemoglobinization [23,28]. On the other hand, in particular in K562 cells, FHC might play other roles besides the control of iron metabolism; it has been proposed that FHC, in its nuclear form, represses β-globin transcription [30], while its silencing modulates the expression of a repertoire of genes during hemin treatment [28]. Collectively, these data suggest that FHC might play multiple and key roles in erythropoiesis, however the underlying mechanisms are not fully elucidated.

### 2.1. Ferritin Heavy Subunit (FHC) Knockdown Negatively Regulates the Erythroid Fate of K562 Cells

**Figure 1:**
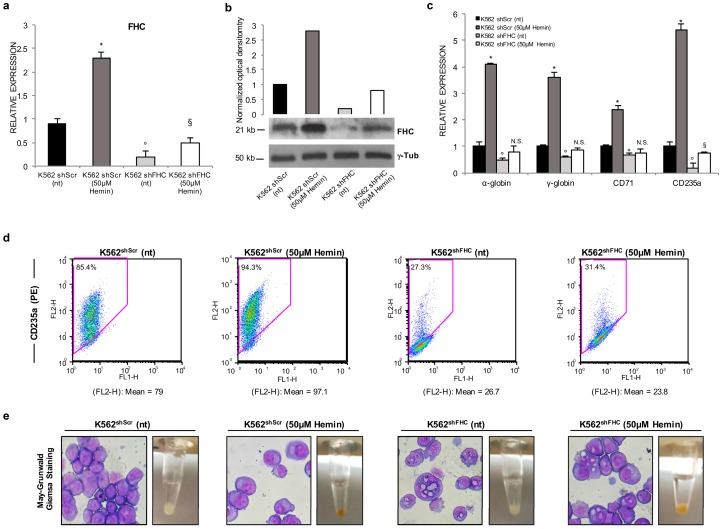
FHC silencing inhibits hemin-induced erythroid differentiation of K562 cells. (**a**) qRT-PCR and (**b**) Western Blot analyses of FHC mRNA and protein levels, respectively, in the control K562^shScr^ and FHC-silenced K562^shFHC^ cells untreated (nt) and treated with 50 µM hemin for 48 h; (**c**) Relative expression of *α-globin*, *γ-globin*, *CD71* and *CD235a* of K562^shScr^ and K562^shFHC^ cells untreated or treated with hemin. All data represent mean ± SD (*n* = 3). * *p* < 0.05 compared with K562^shScr^ cells, ° *p* < 0.05 compared with K562^shScr^ cells, ^§^ *p* < 0.05 compared with K562^shFHC^ cells, N.S.: not significant; (**d**) Flow cytometry analysis of CD235a-positive cells in K562^shScr^ and K562^shFHC^ cells untreated or treated with hemin. Data are reported both as mean fluorescence intensity of CD235a (FL2-H) and as percentage (%) of CD235^+^ cells; (**e**) May-Grünwald-Giemsa staining of K562^shScr^ and K562^shFHC^ cell untreated or treated with hemin.

We have previously found that FHC silencing strongly reduces the hemin-mediated induction of γ-globin synthesis in K562 cells [28]. Here, in order to more clearly define the role of the ferritin heavy subunit in K562 erythroid differentiation we extended the analysis to key molecules associated with erythroid development and functions, such as α-globin, transferrin receptor-1 (CD71), and the glycophorin A (CD235a) erythroid-lineage specific marker. Pools of stably FHC-silenced (K562^shFHC^) and of control cells (K562^shScr^), as previously described [24,28], were treated with 50µM hemin for 48 h. The FHC amounts, evaluated at both mRNA and protein levels, of hemin-treated and untreated K562^shFHC^ and K562^shScr^ cells are shown in Figure 1a,b, respectively. FHC was significantly downregulated in the K562^shFHC^ cells as compared to the control K562^shScr^ cells at both mRNA and protein levels; hemin treatment consistently increased FHC amounts both in the control K562^shScr(50 µM hemin)^ and in the K562^shFHC(50 µM hemin)^ cells when compared to their relative untreated cells (Figure 1a,b). As expected, hemin treatment strongly up-modulated the expression of *α-globin*, *γ-globin*, *CD71*, and *CD235a* mRNAs in K562^shScr^ control cells (Figure 1c). Notably, FHC-silencing was able, per se, to significantly reduce the expression of all four markers; moreover, FHC-silencing almost completely counteracted the effects of hemin treatment and effectively inhibited the induction of *α-globin*, *γ-globin* and *CD71* (Figure 1c). *CD235a* displayed a relatively different behaviour since its mRNA was the most sensitive to FHC knockdown and, in parallel, the only one still responsive to hemin treatment in FHC-silenced cells. We also performed fluorescence-activated cell sorting (FACS) analysis of CD235a in FHC-silenced and control cells, treated and untreated with hemin. FHC knockdown substantially reduced the CD235a positive cells as shown by the substantially lower values of both the mean fluorescence intensity of CD235a and the percentage of CD235a-positive cells in Figure 1d (FLH-2 mean: 79 vs. 26.7; % CD235a^+^: 85.4 vs. 27.3); after hemin treatment K562^shScr^ became nearly completely positive (FLH-2 mean: 97.1; % CD235a^+^: 94.3) while the CD235a^+^ K562^shFHC^ appeared almost completely unaffected (FLH-2 mean: 23.8; % CD235a^+^: 31.4). Along with the modifications of marker expression, microscopic analyses highlighted considerable alterations in the morphology of K562^shFHC^ cells as assessed by May-Grünwald-Giemsa staining, which included features typically not associated to an erythroid phenotype such as increased size, vacuole-rich cytoplasm, and polylobulated nuclei (Figure 1e). After 48 h of culture in the presence of hemin, K562^shScr^ cells showed a globular shape morphology with round nuclei eccentrically located and the cell pellet developed a distinctive red color; conversely, no morphological changes have been observed in K562^shFHC^ cells and the cell pellets became barely pink upon hemin treatment.

**Figure 2:**
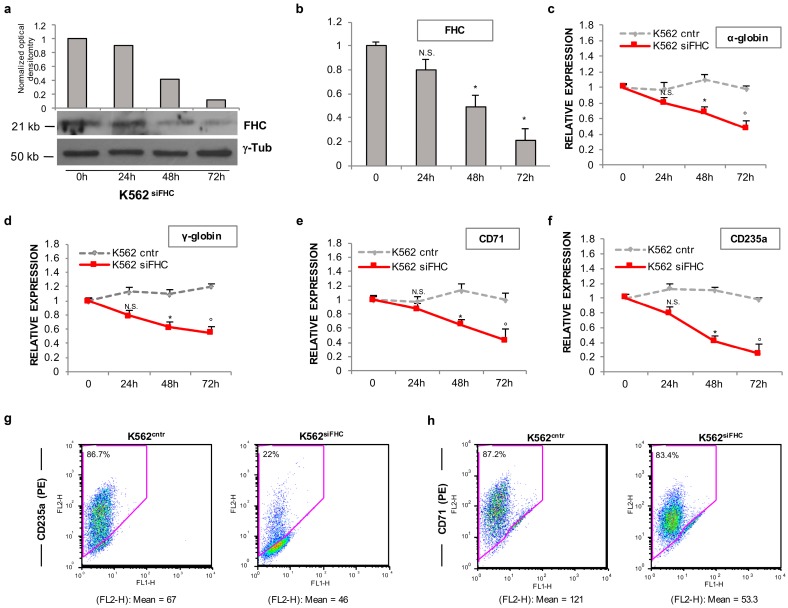
Transient FHC silencing down-regulates erythroid markers expression in K562 cells. FHC amounts of K562 cells at 0, 24, 48 and 72 h after a transient transfection of a specific FHC siRNA at protein (**a**) and mRNA (**b**) levels; Data represent mean ± SD (*n* = 3). * *p* < 0.05 compared with 0 h, N.S.: not significant. The relative expression of *α-globin* (**c**); *γ-globin* (**d**); *CD71* (**e**) and *CD235a* (**f**) in K562^siFHC^ cells at each time point was measured using qRT-PCR. Data represent mean ± SD (*n* = 3). * *p* < 0.05 compared with K562^cntr^ at 48 h; ° *p* < 0.05 as compared with K562^cntr^ at 72 h; N.S.: not significant. Flow cytometry analysis of CD235a- (**g**) and CD71- (**h**) positive cells in K562^cntr^ and K562^siFHC^ cells at 72 h upon FHC siRNA transfection. Data are reported both as mean fluorescence intensity of CD235a and CD71 (FL2-H) and as percentage (%) of CD235^+^ and CD71^+^ cells.

To rule out possible off-target effects of the shRNA, we investigated the expression of *α-globin*, *γ-globin*, *CD71* and *CD235a* in K562 cells where FHC expression was transiently silenced by using a specific FHC-siRNA. Figure 2a shows a representative Western Blot of FHC protein levels at 24 h, 48 h and 72 h after the siRNA transfection while in Figure 2b H-ferritin mRNA levels are represented as the mean of three independent experiments. Consistently with the findings obtained in the stably FHC-silenced cells, the qRT-PCR analyses, shown in Figure 2c–f, highlighted a reduction of the erythroid-specific markers upon transient FHC silencing at each time point with a statistical significance at 48 h and 72 h (*p* < 0.05). Also in these set of experiments *CD235a* showed the highest responsiveness to FHC knockdown. CD235a and CD71 were further investigated, 72 h upon FHC transient silencing, at the cell surface level: Figure 2g–h report representative FACS analyses that highlight markedly reduced levels of both markers in K562^siFHC^ cells compared to the control ones (CD235a, FL2-H mean: 46 vs. 67; % CD235a^+^: 22 vs. 86.7) (CD71, FL2-H mean: 53.3 vs. 121, CD71^+^: 83.4 vs. 87.2).

Taken together, these results indicate that ferritin heavy subunit knockdown is able to strongly inhibit the erythroid transcriptional program in K562 cells, and to almost completely abrogate the transcriptional induction of these genes in response to hemin treatment, thus suggesting that appropriate intracellular amounts of FHC are needed for the correct expression of these key genes.

**Figure 3:**
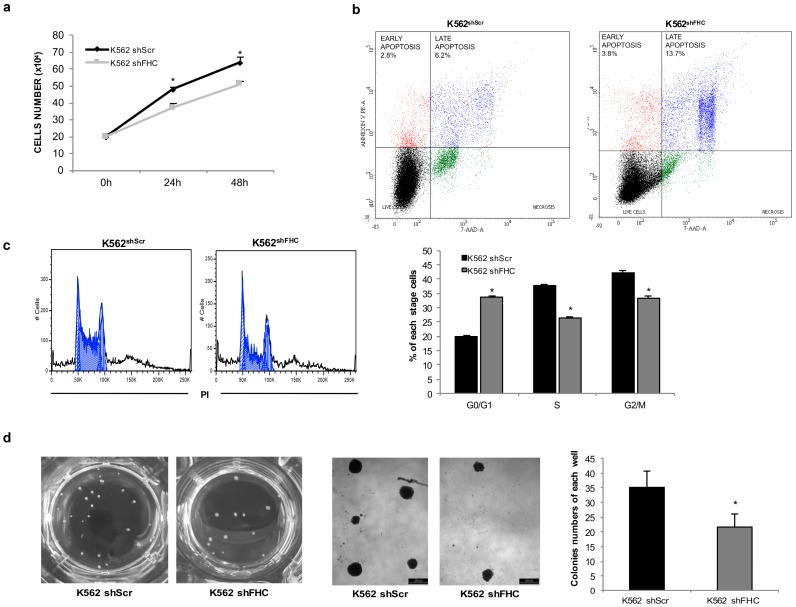
FHC silencing suppresses the proliferative rate and the clonogenic potential of K562 cells. (**a**) Direct cell counting of K562^shScr^ and K562^shFHC^ at 0, 24 and 48 h; (**b**)Apoptosis analysis in K562^shScr^ and K562^shFHC^ using Annexin V/7-AAD double staining. The reported plots are representative of two independent experiments; (**c**) Representative flow cytometry plots of cell cycle analysis in K562^shScr^ and K562^shFHC^ with statistics on the right side. All data represent mean ± SD (*n* = 3). * *p* < 0.05 compared with K562^shScr^ cells; (**d**) Representative microscopy images and statistics of colony formation assay in K562^shScr^ and K562^shFHC^; colonies were observed and counted in each well of 12-well plate. Scale bar: 200µm. All data represent mean ± SD (*n* = 3). * *p* < 0.05 compared with K562^shScr^ cells.

We recently found that FHC knockdown restrains K562 cell proliferation [24]. Because the proliferation rate of a given cell population can be correlated to its differentiation state, here, by using direct cell counting assays, we re-confirmed the decline of K562^shFHC^ proliferative rate in comparison with control cells at 24 h (37 × 10^4^ vs. 48 × 10^4^) and 48 h (51 × 10^4^ vs. 64 × 10^4^) (Figure 3a). Using flow cytometry and Annexin V/7-AAD double staining, we assayed apoptosis of K562^shScr^ and K562^shFHC^ cells and found that FHC silencing slightly increased late apoptosis (Annexin V^+^/7-AAD^+^, 6.2% vs. 13.7%, *p* < 0.05), but not early apoptosis (Annexin V^+^/7-AAD^−^, 2.8% vs. 3.8%) (Figure 3b). The analysis of cell cycle, using PI staining and flow cytometric detection, further confirmed the presence of a higher percentage of K562^shFHC^ cells in the G0/G1 phase as compared to control K562^shScr^ cells (33.6% vs. 19.9%, *p* < 0.05) (Figure 3c). The moderate extent of the FHC silencing-induced G0/G1 cell cycle arrest, in comparison with the strong interference with the erythroid commitment, makes it difficult to correlate these two phenomena. Proliferation and differentiation can either be coupled or inversely correlated in function on the differentiation stage [31], thus further studies are needed to deeply investigate this point. Finally, through clonogenic assays we found that K562^shFHC^ cells displayed an approximately 2-fold decrease of clonogenic potential as compared to the control cells, as shown by representative microscopy images and quantified in the histogram (*p* < 0.05) (Figure 3d).

### 2.2. FHC Effects on K562 Erythroid Fate Are Mediated by miR-150

Transcription factors play a pivotal role in the cell differentiation fate since they are responsible for the expression of lineage-specific genes and for the parallel repression of stemness-related markers [7,32]. In erythropoiesis, the precise balance of GATAs transcription factors is one of the best-known examples [7]. GATA-2 overexpression, in the presence of a reduction of GATA-1, drives megakaryocytic differentiation at the expense of the erythroid one; on the contrary, terminal erythroid differentiation is associated with GATA-1 increase, that in turn, down-regulates GATA-2 expression [6,7].

However, transcription factors alone cannot account for every aspect of haematopoietic differentiation. Post-transcriptional regulatory mechanisms, such as those played by microRNAs, ensure a finer tuning and a more rapid response to stimuli [8,9]. Many recent studies have shown that a number of miRNAs are involved in the fine regulation of the haematopoietic stem cells differentiation, such as miR-15/16, miR-222, miR-150, miR-451, miR-210, and let-7d [8,9]. miR-150, in particular, has been implicated in the control of lineage choice in MEPs fate decision: high levels of miR-150 drive MEPs toward megakaryocytic maturation, whereas low levels lead to erythroid commitment [12,18].

Recently, we have demonstrated that FHC controls the expression of a set of miRNAs in a variety of cancer cell types, including K562 cells [24] and that, in SKOV-3 ovarian carcinoma cells, FHC promotes the expansion of a subset of cells with stem-like features through the modulation of miR-150 [27].

**Figure 4:**
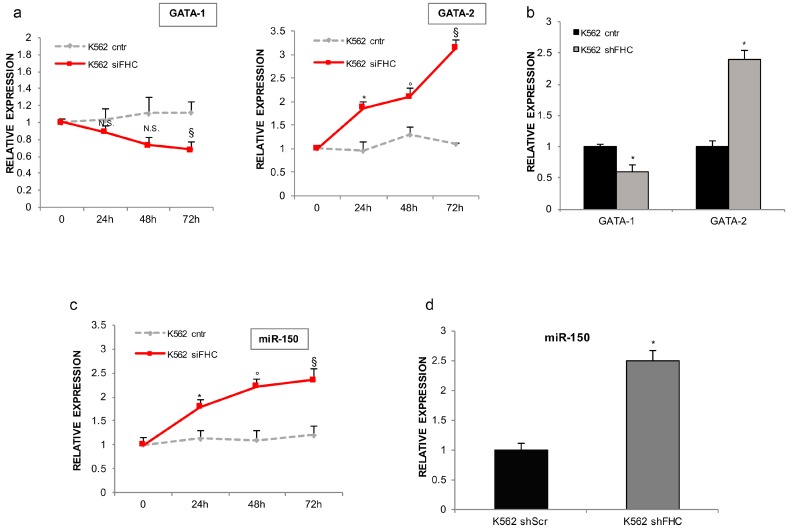
FHC silencing reduces GATA-1 and induces miR-150 up-regulation. (a) qRT-PCR analysis of *GATA-1* and *GATA-2* genes in transiently FHC-silenced K562^siFHC^ cells at 0 h, 24 h, 48 h, and 72 h, compared to control K562^cntr^ cells. All data represent mean ± SD (*n* = 3). * *p* < 0.05 compared with K562^cntr^ at 24 h; ° *p* < 0.05 compared with K562^cntr^ at 48 h; ^§^ *p* < 0.05 as compared with K562^cntr^ at 72 h. N.S. not significant; (**b**) qRT-PCR analysis of *GATA-1* and *GATA-2* genes in stably FHC-silenced K562^shFHC^ cells compared to control K562^shScr^ cells. All data represent mean ± SD (*n* = 3). * *p* < 0.05 compared with K562^shScr^ cells; (**c**) TaqMan analysis of miR-150 in transiently FHC-silenced K562^siFHC^ cells at 0 h, 24 h, 48 h, and 72 h, compared to control K562^cntr^ cells. All data represent mean ± SD (*n* = 3). * *p* < 0.05 compared with K562^cntr^ at 24 h; ° *p* < 0.05 compared with K562^cntr^ at 48 h; ^§^ *p* < 0.05 compared with K562^cntr^ at 72 h; (**d**) TaqMan analysis of miR-150 in stably FHC-silenced K562^shFHC^ cells compared to control K562^shScr^ cells. All data represent mean ± SD (*n* = 3). * *p* < 0.05 compared with K562^shScr^ cells.

In order to establish whether modulation of miR-150 expression in response to FHC knockdown may contribute to the phenotypes observed in K562 and described above, we analyzed the steady state amounts of *GATA-1* and *GATA-2* mRNAs and those of miR-150 in both transiently and stably FHC-silenced cells. As shown in Figure 4a,b, FHC silencing down-regulates *GATA-1* and, at the same time, strongly induces *GATA-2* expression. As highlighted in Figure 4c,d, this is accompanied by a significant increase in miR-150 levels upon both transient and stable FHC silencing. Notably, both *GATAs* switch and miR-150 up-regulation are consistent with the FHC-mediated arrest of K562 erythroid differentiation.

**Figure 5:**
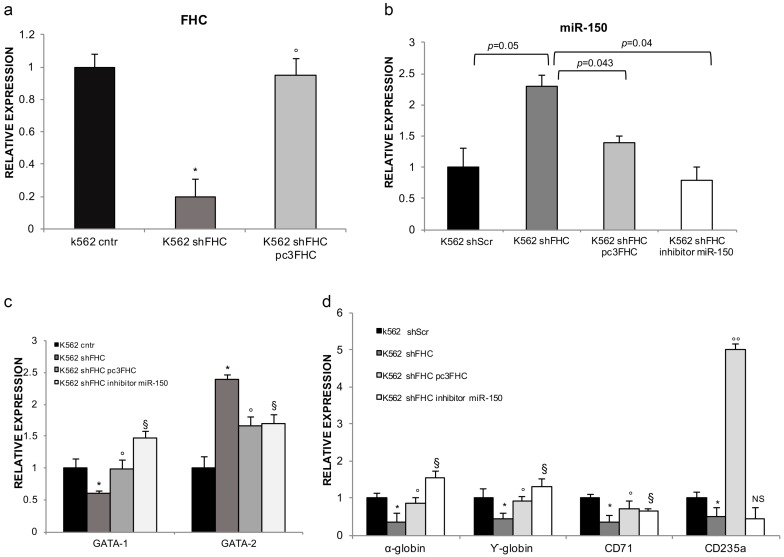
miR-150 mediates the control exerted by FHC on *GATAs*, *α-globin*, *γ-globin* and *CD71* expression. (**a**) pc_3_FHC expression vector almost completely restore FHC amounts in FHC-silenced K562 cells (K562^shFHC/pc3FHC^). Data represent mean ± SD of 3 qRT-PCR analysis. * *p* < 0.05 compared with K562^shScr^; ° *p* < 0.05 compared with K562^shFHC^; (**b**) TaqMan analysis of miR-150 levels after FHC reconstitution and miR-150 inhibition highlight a significant down-regulation in K562^shFHC/pc3FHC^ and K562^shFHC/miR−150 inhibitor^ compared to K562^shFHC^ cells; (**c**) qRT-PCR analysis of *GATA-1* and *GATA-2* genes in K562^shFHC/pc3FHC^ and K562^shFHC/miR−150 inhibitor^ as compared to K562^shFHC^ cells. Data represent mean ± SD (*n* = 3). * *p* < 0.05 compared with K562^shScr^ at; ° *p* < 0.05 compared with K562^shFHC^; ^§^ *p* < 0.05 compared with K562^shFHC^; (**d**) qRT-PCR analysis of *α-globin*, *γ-globin*, *CD71* and *CD235a* genes in K562^shFHC/pc3FHC^ and K562^shFHC/miR−150 inhibitor^ compared to K562^shFHC^ cells. Data represent mean ± SD (*n* = 3). * *p* < 0.05 compared with K562^shScr^ at; ° *p*< 0.05 when compared with K562^shFHC^; °° *p* < 0.001 compared with K562^shFHC^; ^§^ *p* < 0.05 compared with K562^shFHC^; N.S. not significant.

To shed further light on the role of miR-150 in the cascade of molecular events induced by FHC silencing, we either reconstituted FHC or inhibited miR-150 inK562^shFHC^ cells. FHC reconstitution, whose efficiency is shown in Figure 5a, determined a significant reduction of miR-150, thus confirming the existence of an inverse correlation between FHC and miR-150 steady state amounts (Figure 5b). The results of a triplicate set of experiments, graphically represented in Figure 5c, indicate that both FHC reconstitution and miR-150 inhibition were able to revert the *GATAs* switch. This is the first report showing that GATAs transcription factors are potential downstream molecules of miR-150. Since the bioinformatic online prediction tool FINDTAR3 did not underline any complementary regions between *GATAs* and miR-150, we propose the existence of an indirect relationship between these two molecules, mediated by an intermediate still to be determined. The down-modulation of *α-globin*, *γ-globin* and *CD-71* were efficiently counteracted by both FHC reconstitution and miR-150 inhibition (Figure 5d). It has been already reported that the expression of both *globins* and *CD71* is transcriptionally regulated by GATA1 [33,34]. Taken all together our data suggest a model in which FHC amounts modulates, through miR-150, *GATA1* that in turn regulates globins and *CD71*. *CD235a* appears to be not included in this pathways since its amounts strongly responded to the FHC reconstitution, increasing by approximately 5-fold, but were unaffected by miR-150 inhibition.

Notably, in FHC-silenced K562 cells, the up-regulation of miR-150 is unable to promote megakaryocytic differentiation since the analysis of gene and surface expression of CD41, CD61, and CD110 MK-specific markers did not highlight any modification in their steady state amount (Figure S1). Megakaryocytic differentiation is driven not only by miR-150 but also by other microRNAs such as miR-34a, miR-146a, miR-27a, and miR-28 [35]. The natural conclusion of our observations is that, at least in FHC-silenced K562 cells, miR-150 is a central hub in the molecular phenomena resulting in the arrest of the erythroid commitment while the megakaryocytic differentiation requires other so far uncovered regulatory molecules.

The mechanisms by which FHC inhibits miR-150 expression require further and more targeted analyses. To the best of our knowledge, in K562 cells, the nuclear form of H ferritin has been suggested as specific repressor of the β-globin promoter [30] but no further evidences have emerged in this direction. On the other hand, it has been recently shown that the transcript of FTH1P3, one intronless member of the FHC multigene family, acts as molecular sponge of miR-224-5p in oral squamous cell carcinoma cells [36]. Interestingly, as reported in Figure S2, the FINDTAR3 bioinformatic prediction software highlights the existence of two regions of complementarity between FHC mRNA and miR-150 (FHC mRNA NM_002032 positions: 38–73; 165–188). This observation constitutes the molecular ground for future studies aimed at analyzing a possible role of FHC mRNA as competitive endogenous RNA (ceRNA).

In conclusion, we demonstrate that a costant expression of FHC is essential for K562 erythroid differentiation since either its stable or its transient silencing is associated with an arrest toward this commitment. Furthermore, our data delineate a new regulatory axis through which FHC controls erythroid differentiation and lend strong support to the notion that, in addition to its role in iron metabolism, FHC can act as a relevant regulator of gene expression through the modulation of miR-150.

## 3. Materials and Methods

### 3.1. Cell Culture

K562 cells were obtained from the American Type Culture Collection (ATCC, CCL-243; Manassas, VA, USA). K562 cells were grown in RPMI 1640 media (Sigma Aldrich, St. Louis, MO, USA) supplemented with 10% FBS (Thermo Fisher Scientific, Waltham, MA, USA) at 37 °C in 5% CO_2_. Hemin (50 μM) (Sigma Aldrich) was used to induce erythroid differentiation of K562 cells.

### 3.2. K562 Cells Transfection and Transduction

FHC silencing of K562 cells was performed using two different methods: (i) stable transduction of a lentiviral DNA containing an shRNA targeting the 196–210 region of the FHC mRNA (sh29432) (K562^shFHC^) or a control shRNA without significant homology to known human mRNAs (K562^shScr^) and (ii) transient transfection of a pre-cast siRNAspecific for FHC (K562^siFHC^) or a negative control siRNA (K562^cntr^) (Thermo Fisher Scientific). Stable transduction was achieved, as previously reported [19,23]. Transient transfections were performed using the Amaxa Nucleofactor kit (Lonza, Basel, Switzerland) [37]. FHC stable knock-down was verified by Western Blot analysis and RT-qPCR while FHC transient knockdown was checked at 24, 48 and 72 h by RT-qPCR analysis. FHC-reconstitution in K562^shFHC^ cells was performed using the expression vector containing the full length of human FHC cDNA (pc_3_FHC) (K562^shFHCpc^_3_^FHC^).

K562^shFHC^ cells were also transiently transfected with a final concentration of 100 nM of a specific miR-150 inhibitor (K562^shFHCmiR−150 inhibitor^) obtained from Thermo Fisher Scientific, using the Amaxa Nucleofactor kit (Lonza). 48 h after transfection, miR-150 levels were measured by TaqMan microRNA Assay (Thermo Fisher Scientific).

### 3.3. RNA Extraction and Quantitative Real-Time PCR (qRT-PCR)

Total RNA was extracted from K562 cells with the TRizol RNA isolation method (Thermo Fisher Scientific), according to the manufacturer’s instructions. The purity and the integrity of each RNA sample were checked, as previously reported [26]. Then, 1 μg of RNA from each sample was reverse transcribed by using High Capacity cDNA Reverse Transcription Kit (Thermo Fisher Scientific). Gene expression analysis was performed by using SYBR^®^Green qPCR Master Mix (Thermo Fisher Scientific) [24]. Primers to detect *FHC*, *CD235a*, *CD71*, *α-globin*, *γ-globin*, *GATA-1* and *GATA-2* are reported in Table 1. Relative quantification between samples and control transcript levels was performed by using the comparative 2^−∆∆*C*t^ method [26]. Each sample was normalized to its glyceraldehyde 3-phosphate dehydrogenase (*GAPDH*) content.

### 3.4. TaqMan miR-150 Analysis

Specific cDNA synthesis for miR-150, was performed using TaqMan^®^ MicroRNA Reverse Transcription Kit (Thermo Fisher Scientific) containing microRNA-specific RT primers and Taqman miRNA assay. To measure miRNAs expression levels, 1.33 µL of each cDNA was added to the specific TaqMan microRNA Assay (20×) and TaqMan 2× Universal PCR Master MiX (Thermo Fisher Scientific). The amplification conditions for miRNA expression profile were the following: 10 min at 95 °C, 40 cycles at 95 °C for 15 s, and 60 °C for 60 s. The experiments were performed in duplicate and the analysis was performed using the2^−∆∆*C*t^ formula using snRNA U6 as housekeeping microRNA [24].

### 3.5. Western Blot Analysis

Whole-cell lysis, protein extraction and Western Blot analyses of cultured K562^shScr^, K562^shFHC^, K562^cntr^, and K562^siFHC^ cells were performed, as previously reported [25,26]. For FHC protein quantification, the incubation of the anti-rabbit polyclonal primary anti-FHC (H-53) (1:200; sc-25617, Santa Cruz Biotechnology, Dallas, TX, USA) followed by the incubation with the HRP-conjugated secondary antibody (1:3000 Cell Signaling) was carried out. The goat polyclonal anti-γ-Tubulin antibody (C-20) (1:3000; sc-7396, Santa Cruz Biotechnology) was used as loading control. The immunoreactive bands were visualized with the ECL Western blotting detection system (Santa Cruz Biotechnology) and the bands intensity was quantified by using ImageJ (National Institutes of Health, Bethesda, MD, USA).

### 3.6. Flow Cytometry Analyses

The expression of erythroid-specific cell-surface markers was determined by direct immunofluorescence using the following conjugated antibodies: PE-CD235a and PE-CD71 (Miltenyi Biotec, Bergisch Gladbach, Germany). Briefly, 2 × 10^5^ cells were suspended in 100 μL PBS supplemented and then stained with fluorochrome-conjugated antibodies for 30 min on ice. Cells were washed twice with PBS before analysis. Flow cytometry analyses were carried out 1 h after staining. The analysis was performed using FACScan flow cytometry (Becton Dickinson, Franklin Lakes, NJ, USA) and the data files were analyzed by FlowJo software (FlowJo v8.8.6, Becton Dickinson, Franklin Lakes, NJ, USA).

For cell cycle analysis, 5 × 10^5^ cells were washed in PBS twice, fixed by adding ethanol 100%, in a drop wise manner, and then was stored at −20 °C overnight. Cells were then rinsed twice to remove ethanol and incubated with PI solution for 1 h, in the dark, at room temperature prior to FACS analysis.

Apoptosis analysis was carried out using the Annexin V-PE Apoptosis Detection Kit (Becton Dickinson, Franklin Lakes, NJ, USA). Cells were washed twice with cold PBS and then resuspended in 1× Binding Buffer at a concentration of 1 × 10^6^ cells/mL. Then, 5 µL of PE Annexin V and 5 µL 7-AAD were added to the cells incubated for 15 min at room temperature in the dark. Finally, 400 µL of 1× Binding Buffer was added to each sample before flow cytometry analysis. The analysis was performed in duplicate; here, a representative plot has been reported.

### 3.7. Direct Cell Counting

Briefly, 20 × 10^4^ cells/well were seeded during the exponential phase of proliferating cells. Cell pellets, obtained by centrifugation at 1000 rpm × 5 min, were washed with fresh PBS and then resuspended in 5 mL by vigorously pipetting to disperse any clumps. The cell count was performed by mixing 50 μL of sample with 50 μL of 0.4% trypan blue solution, afterwards the mixture was loaded into the Bürker chamber. Each cell-count was performed in triplicate by using a 10× objective according to the standard methods.

### 3.8. Haematopoietic Colony Formation Assay

For colony formation assays, K562 cells (5 × 10^2^/mL) were plated in tissue culture 12 well plates in MethoCult H4100 medium (StemCell Technologies, Vancouver, BC, Canada) consisting of RPMI 1640 supplemented with 1% methylcellulose and 10% FBS. After 14 days, plates were scored for colony forming units (CFUs) using an inverted microscope (Leica, Wetzlar, Germany).

### 3.9. Cell Morphology Assay

For evaluating cell morphology, cells were cytocentrifuged onto glass slides, fixed in methanol and stained with May Grunwald-Giemsa (Thermo Fisher Scientific) and photographed with 40× magnification with a digital camera Leica DFC420 C and Leica Application Suite Software (v1.9.0, Leica).

### 3.10. Statistical Analysis

Statistical significance of data was assessed by Student’s *t*-test. *p* values < 0.05 were considered significant.

### 3.11. Bioinformatic Analysis

We used the publicly available software programs FINDTAR3 to predict the existence of complementary regions between miRNAs and mRNAs based on seed-pairing, free energy of miRNA:mRNA duplex, and proper dynamic programming score.

## Figures and Tables

**Table 1:** Primers used in qRT-PCR analysis.

Gene	Forward Primer	Reverse Primer
*GAPDH*	5′-TGA TGA CAT CAA GAA GGT GGT GAA G-3′	5′-TCC TTG GAG GCC ATG TGG GCC AT-3′
*FHC*	5′-CAT CAA CCG CCA GAT CAA C-3′	5′-GAT GGC TTT CAC CTG CTC AT-3′
*α-globin*	5′-GTG GAC GAC ATG CCC AAC-3′	5′-TAT TTG GAG GTC AGC ACG GT-3′
*γ-globin*	5′-CAG AAA TAC ACA TAC ACA CTT CC-3′	5′-GAG AGA TCA CAC ATG ATT TTC TT-3′
*CD71*	5′-ACT GGT CCA TGC TAA TTT TGG T-3′	5′-AGT TCT GCG TTA ACA ATG GGA-3′
*CD235a*	5′-GAG AAA GGG TAC AAC TTG CC-3′	5′-CAT TGA TCA CTT GTC TCT GG-3′
*GATA-1*	5′-GAT GAA TGG GCA GAA CAG GC-3′	5′-TAG CTT GTA GTA GAG GCC GC-3′
*GATA-2*	5′-GAA CCG ACC ACT CAT CAA GC-3′	5′-GCA GCT TGT AGT AGA GGC CA-3′

## Supplementary Materials

The following are available online at www.mdpi.com/1422-0067/18/10/2167/s1.
