# Supplementary material for: Ferritin Heavy Subunit Silencing Blocks the Erythroid Commitment of K562 Cells via miR-150 up-Regulation and GATA-1 Repression
Source: Int J Mol Sci. 2017 Oct 17;18(10):2167. doi: 10.3390/ijms18102167 (PMC5666848; doi:10.3390/ijms18102167)
Supplement: Supplementary file 1 [file ijms-18-02167-s001.pdf]

Supplementary Materials

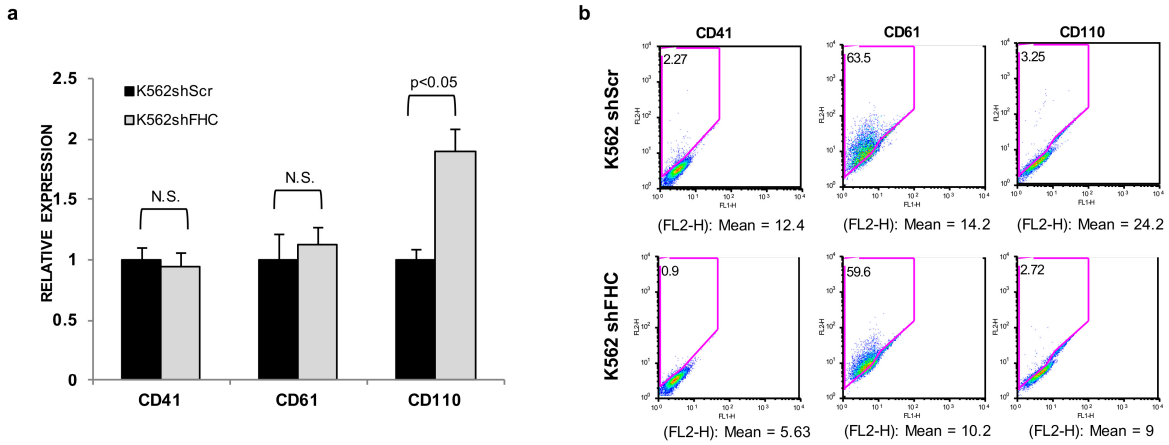

Figure S1. FHC silencing doesn't affect the expression of megakaryocytic markers. (**a**) Relative expression of *CD41, CD61* and *CD110* of K562^{shScr} and K562^{shFHC} cells. Data represent mean ± SD (*n*=2). N.S.: not significant; (**b**) Representative plots of two independent flow cytometry analyses of CD41, CD61 and CD110 surface expression in K562^{shScr} and K562^{shFHC} cells. Data are reported both as mean fluorescence intensity (FL2-H) and as percentage (%) of CD41^{+}, CD61^{+} and CD110^{+} cells.

<table>
<tr><th>miRNA</th><th>mRNA</th><th>Position</th><th>Structure</th><th>Loop Score</th><th>ΔG</th><th>Recommendation</th></tr>
<tr><td>hsa-miR-150-5p</td><td>NM_002032</td><td>38-73</td><td>3' GUGACCA--UGUUCC------------CAACCCUCU 5'
|||||*|||||************||||||:**
5' CCCTGGTCACCAAGGCAGTGCATGCATGTTGGGGTT 3'</td><td>20.00</td><td>-25.00</td><td>excellent</td></tr>
<tr><td>hsa-miR-150-5p</td><td>NM_002032</td><td>165-188</td><td>3' GUGACCAUGUUCC-CAACCCUCU 5'
*********:|||**||||||**
5' GTTGTCTTTGAGGTCTTGGGATG 3'</td><td>20.00</td><td>-16.60</td><td>excellent</td></tr>
</table>

Figure S2. FHC mRNA-miR-150 complementary regions. FINDTAR3 online tool predicted two putative complementary regions between FHC mRNA and miR-150 (FHC mRNA (NM_002032) positions: 38–73; 165–188).
